# Risk perceptions regarding inclusion of seasonal influenza vaccinations in the school immunization program in Israel: Arab vs. Jewish mothers

**DOI:** 10.1371/journal.pone.0267279

**Published:** 2022-04-18

**Authors:** Nour Abed Elhadi Shahbari, Anat Gesser-Edelsburg, Nadav Davidovitch, Shuli Brammli-Greenberg, Gustavo S. Mesch

**Affiliations:** 1 School of Public Health, University of Haifa, Haifa, Israel; 2 The Health and Risk Communication Lab, University of Haifa, Haifa, Israel; 3 Department of Health Systems Management, Faculty of Health Sciences, Ben Gurion University of The Negev, Beer Sheva, Israel; 4 Braun School of Public Health and Community Medicine, The Hebrew University of Jerusalem, Jerusalem, Israel; 5 Department of Sociology and Anthropology, University of Haifa, Haifa, Israel; Uniwersytet Zielonogorski, POLAND

## Abstract

**Background:**

The issue of whether to include seasonal influenza vaccinations in school-located vaccination programs (SLIV) has been examined in many countries, mainly in the context of economic effectiveness and morbidity prevention. Yet not enough studies have examined the impact of parental risk perceptions, health literacy and SLIV on parental vaccination uptake.

**Objectives:**

The most recent statistics in Israel point to a higher rate of seasonal influenza vaccination among Arab children (aged 7–9 years) than among Jewish children in the same age group. The present study attempts to explain this high vaccination uptake among mothers from Arab society by comparing their risk perceptions regarding seasonal influenza vaccination and disease to those of Jewish mothers. The study further examines the impact of SLIV on parental risk perceptions and influenza vaccination uptake.

**Methods:**

This cross-sectional study included mothers of children in the second and third grades faced with the decision of whether their children should receive the seasonal influenza vaccination at school. The study population included a stratified sample of Jewish mothers (n = 159) and Arab mothers from all the Arab population sub-groups: Muslim, Christian, Druse and Bedouin (n = 534).

**Results:**

A comparison of the Arab and Jewish populations revealed a significant difference in vaccination rates; 61.7% among Arab mothers compared to 33.5% among Jewish mothers (χ^2^(1) = 39.15, P<0.0001). Moreover, significant differences emerged between the Arab and Jewish populations in health literacy and ability to seek information regarding the seasonal influenza vaccination (t (691) = -5.81, p < 0.0001). While no differences emerged in mothers’ perceptions regarding influenza as a disease (t (691) = 1.20, p = 0.2318), Arab mothers perceived the vaccination to be safer than Jewish mothers (t (691) = 2.74, p = 0.0063) and saw its inclusion in the school-located vaccination program as providing more legitimacy (Z = -6.6719, P < .0001).

**Conclusion:**

This study showed that the factors influencing vaccination uptake among both the Arab and the Jewish populations include perceived influenza risk, perceived vaccination risk, inclusion in the school-located vaccination program and health literacy. Moreover, influenza vaccination uptake is higher among those who have positive attitudes toward vaccinations, low risk perceptions regarding the vaccine, and low health literacy that impedes their ability to seek information. The research also points to the need for education and tools to boost health literacy among minority groups so that mothers can make independent and informed decisions about whether or not to vaccinate their children.

## Introduction

### Seasonal influenza

Influenza is a contagious respiratory disease that usually occurs during the period from the end of autumn to the beginning of spring [1]. One unique attribute of the influenza virus is that its genetic clades change every year [2]. The influenza virus can cause mild to severe illness, particularly among people at risk: children under age 2, pregnant women (and up to two weeks after giving birth), people age 65 and over and those with chronic illnesses [3]. The complications of influenza can be physiological (side effects) [4], epidemiological [5] and economic [6]. From an epidemiological perspective, the burden of seasonal influenza morbidity can be seen in health system statistics, such as number of hospitalizations and morbidity level.

### Effectiveness of seasonal influenza vaccination among children

The effectiveness of seasonal influenza vaccination is measured by its health and economic consequences. While research has shown that vaccination is an economical, effective and equitable means of preventing the flu each year [7], there is still some controversy surrounding its success. Moreover, the vaccine’s effectiveness can change from year to year [8]. Several studies and systematic reviews have pointed to varying effectiveness according to age group [9, 10]. A meta-analysis published in 2020 showed that influenza vaccination offers significant protection against any influenza-related pediatric hospitalization. The meta-analysis confirmed the importance of a full vaccination strategy in this pediatric population, while also underscoring the significant protection the influenza vaccine offers children under the age of five, an age group at high risk for influenza-related complications such as hospitalization [11]. On the other hand, a review from the Cochrane database showed that the absolute reduction in influenza and ILI (Influenza-like illness) among children varied considerably across different study populations, making it difficult to predict how these findings translate to different settings [12].

### Policies and regulations for administering the influenza vaccine at school

Since 2008, the United States Advisory Committee on Immunization Practices (ACIP) has recommended universal annual seasonal influenza vaccination for all children between ages 6 months and 18 years [13]. Other nations also recommend vaccinating children against the flu [14]. Nevertheless, many countries have no such regulations, based on the common belief that influenza vaccinations are not effective for children under the age of five and on the lack of sufficient cost/benefit evidence regarding vaccination of school-aged children [15]. Indeed, most countries only recommend influenza vaccination for children who are at high risk due to pre-existing conditions [16].

Inclusion of the influenza vaccination in school-located vaccination programs is designed to provide a positive framework for vaccinations in general and thus make parents more compliant [17]. The health system also takes other issues into consideration, among them making the vaccine accessible to weakened population groups [18], reducing the need to burden parents and children with additional medical visits [13], and generating herd immunity in the general population [19].

Evidence regarding the effectiveness of school influenza vaccinations varies. On the one hand, recent randomized controlled trials (RCTs) of school-located influenza vaccination (SLIV) in a New York suburban area during the 2009–2010, 2010–2011, 2014–2015, and 2015–2016 flu seasons found that SLIV increased overall influenza vaccination rates by 5 to 16 percentage points among elementary school children and 5 percentage points among secondary school children [13]. In addition, the results of the British school-located vaccination program show a decline in influenza cases among children and in the general population [20]. On the other hand, in 2015 the rates of influenza vaccination among schoolchildren in Los Angeles remained low [21], and other studies showed that giving the influenza vaccine at school does not decrease the contagion rate for seasonal influenza among schoolchildren or among the general population [22].

### Vaccination rates in Israel before and after the influenza vaccine was included in the school-located vaccination program

Beginning in 2016, one dose of inactivated influenza vaccine were administered to all second graders in Israel through the Ministry of Health’s School Health Services. In 2017, third graders were also included in this program, and starting in September 2019, fourth graders were also included, such that during the 2019–2020 flu season, the vaccine was given to second, third and fourth grade pupils. The pupils were vaccinated at school during school hours by the school nurse. Vaccination is not mandatory, requires parental permission and is given free of charge [23]. According to figures from the Israel Center for Disease Control (ICDC), in the winter of 2018/2019 about 21% of the general population of Israel took the flu vaccination, while the vaccination rate among children aged 6 months to 5 years was about 26% [24]. Yet no data were published regarding vaccination rates among children aged 7–9 years group before the vaccine was included in the school-located program, either for the Arab or for the Jewish populations.

In 2015, even before the influenza vaccine was included in the school-located vaccine program, influenza vaccination rates among Israel’s Muslim population (representing 97% of Israel’s Arab population) had reached 45% [25]. In 2016–2017, after the seasonal flu vaccination began being offered at school, the vaccination rate for second graders in the Arab population reached 84%, compared to 47% in the Jewish population. The Ministry of Health 2019 vaccination report reveals major differences in vaccination coverage between the two populations, with an 81.4% vaccination rate in the Arab schools compared to 44–54% in Jewish schools. The main reason for not giving the vaccination was parental refusal [26].

### Factors affecting parents’ decision to vaccinate their children against the influenza

Many studies have attempted to understand the factors influencing decision-making with respect to vaccinations in general. Socioeconomic attributes are among the major factors [27]. Some studies show that educated people tend to immunize their children less than those with less education [28], while others show that educated people tend to immunize their children more [29]. Factors such as culture [30], source of information about the vaccine [31] and physician recommendation [32] also influence vaccination decision-making. Trust in government is also a factor, such that mothers who place more trust in the government tend to exhibit greater vaccination compliance [28]. These variables also apply to making decisions about seasonal influenza vaccinations [33]. Other components are related to people’s perceptions of the seriousness of the illness relative to the vaccine’s side effects [25, 32, 34] and to the social group’s attitude toward vaccinations.

Research in other countries has also examined the inclusion of influenza vaccinations in the schools, mainly with respect to economic impact and morbidity prevention in the health system [13]. Yet not enough studies have examined the impact of SLIV on parental risk perceptions regarding influenza severity and vaccine safety. Moreover, because vaccination compliance rates in Israel are higher among the Arab population than among the Jewish population, the impact of SLIV should be examined in both populations and preliminary data regarding SLIV influence should be extended. Hence, this study seeks to compare the illness and vaccination risk perceptions of Arab mothers to those of Jewish mothers and to examine the impact of SLIV on vaccination uptake in these two populations, based on the preliminary data available.

### Research hypotheses

We hypothesize that the following factors will differentially affect seasonal influenza vaccination uptake among the Arab and the Jewish populations: ethnicity and health literacy, perceived disease risk, perceived vaccination risk and SLIV. More specifically:

1. **Impact of ethnicity on seasonal influenza vaccination uptake:** A correlation between ethnicity and seasonal influenza vaccination uptake will be found in both population groups, such that Arab mothers will exhibit a higher vaccination uptake rate than Jewish mothers. Moreover, Arab mothers will exhibit more difficulties with literacy and lower information-seeking abilities than Jewish mothers, such that their rates of seasonal flu vaccination of their children will be higher than those of Jewish mothers.
2. **Risk perceptions regarding seasonal influenza vaccination:** Arab mothers will exhibit more positive attitudes than Jewish mothers toward vaccinations in general and will therefore report lower risk perceptions regarding the seasonal influenza vaccination. Moreover, Jewish mothers will perceive the seasonal influenza vaccination as more dangerous than Arab mothers, such that seasonal flu vaccination rates will be lower among Jewish mothers.
3. **Risk perceptions regarding seasonal influenza as a disease:** Arab mothers will perceive seasonal influenza as a more dangerous disease than Jewish mothers, such that they will exhibit higher rates of compliance to seasonal flu vaccination for their children.
4. **Inclusion of seasonal flu vaccinations in the school-located vaccination program**: SLIV will provide more legitimacy to Arab mothers regarding vaccinating their children than to Jewish mothers.

## Materials and methods

### Research population

This cross-sectional study included mothers of children in the second and third grades who were asked to decide whether their children should receive the seasonal influenza vaccination recently introduced into the school vaccination program.

The study population (Table 1) included a stratified sample of Jewish mothers (n = 159) and Arab mothers from all the Arab population sub-groups: Muslim, Christian, Druse and Bedouin (n = 534).

**Table 1:** Socio-demographic characteristics of Jewish and Arab mothers (n = 693).

Socio-demographic attributes	Category	n (%)
**Age**	25–35	102 (14.72)
	36–40	288 (41.56)
	41–45	215 (31.02)
	46+	88 (12.70)
**Number of Children**	2	95 (13.71)
	3	271 (39.11)
	4	200 (28.86)
	5	87 (12.55)
	6+	40 (5.77)
**Grade level of child receiving vaccination**	Second Grade	185 (28.29)
	Third Grade	229 (35.02)
	Fourth Grade	240 (36.70)
**Mother’s Educational Level**	Elementary School	16 (2.31)
	High School	70 (10.10)
	Post-Secondary	246 (35.50)
	Bachelor’s Degree	169 (24.39)
	Master’s Degree and higher	192 (27.71)
**Degree of Religiosity**	Secular	296 (42.77)
	Traditional	98 (14.16)
	Religious	299 (42.12)

### Sampling method and data collection

Stratified sampling [35] was conducted according to the examined ethnic sub-groups: Muslim Arabs, Christian Arabs, Druse Arabs, Bedouin Arabs and Jews. Each group having an equal number of participants and all participants were mothers of children in the second and third grades. These sub-groups were not sampled according to their relative size in the Israeli population. Rather, each group was considered equal to the other sub-groups in the sample, such that each sample contained an equal number of participants to facilitate group comparisons. The research participants were randomly recruited by means of stratified random sampling [36] at 43 schools in localities in the northern district of Israel with Arab or mixed Arab/Jewish populations. The questionnaires were distributed by hand to the children at school, together with a letter to the parents and a request for their informed consent. The children gave the questionnaires to their mothers and returned them at school the next day. The response rate was 92%. The rationale for choosing mothers was that they are the primary parent in the family when it comes to making decisions about vaccinations [37]. The sampling method was manual rather than via an online questionnaire because a substantial portion of the Arab population, and particularly the Northern Bedouin population, has low digital literacy [38].

### Research instrument

The quantitative questionnaire (S1 Appendix) was based on validated questionnaires from the literature and was culturally adapted to the population in Israel. In building the questionnaire, we conducted a pilot study among representatives of the target population groups. To help us construct the study questionnaire, we also conducted qualitative research by means of personal interviews with mothers of second and third graders who were vaccinated through the SLIV [39]. The study questionnaire included the following parts: 1) A demographic information questionnaire that contained questions about age, number and age of children, education, ethnic origin, and geographic location; 2) a questionnaire on attitudes about vaccinations that included 16 questions taken from the questionnaire by Al-lela, Bahari, Al-abbassi, and Basher (2011) [40]. For each question, respondents ranked their agreement on a Likert scale ranging from 1 (do not agree) to 5 (very much agree). The questionnaire included items such as “All vaccinations recommended by the health authorities are safe” and “When I think about vaccinations, I am concerned about side effects”; 3) a seasonal influenza vaccination questionnaire that included self-report yes/no questions about seasonal influenza vaccination compliance and an open question for explaining why or why not. An additional 32 questions were taken from the questionnaire by Weinstein, Kwitel, McCaul, Magnan, Gerrard and Gibbons (2007) [41] with respect to opinions about the illness, the vaccination and its risks. Participants rated these questions on a Likert scale ranging from 1 (do not agree) to 5 (very much agree). The questionnaire used in the current study also included questions related to searching for information about influenza vaccinations and questions regarding participants’ perspective on school-located vaccination programs based on the findings of a qualitative study by the researchers [39].

### Reliability and validity

As noted, the study questionnaire was based on the questionnaire by Weinstein, Kwitel, McCaul, Magnan, Gerrard and Gibbons (2007) [41], which has a reported internal reliability of α = 0.9. Furthermore, the internal reliability of the vaccination attitudes questionnaire (Al-lela, Bahari, Al-abbassi, & Basher, 2011) was reported as α = 0.54 [40], signifying a high level of internal reliability. After collecting the data, we calculated the Cronbach’s alpha of the current research questionnaire, yielding an internal reliability of α = 0.8. The questions were originally formulated in Hebrew. They were then translated into Arabic by two different translators and cross-checked. In addition, a pilot study was conducted among a limited sample of 80 participants to validate the content and to assess the wording for cultural appropriateness to the target population. After the data were entered into the data file, they underwent quality control to detect any errors in data entry. This quality control entailed observing the range of data for each question and generating distributions. In addition, outliers were examined for the different variables, and the assumption of normal distribution for the variables was also examined. Moreover, to deal with the possibility that each of the factors influenced the dependent variable and the independent variables, during the analysis we measured the correlations between each factor and the dependent and independent variables.

### Ethics

The study was approved by the Ethics Committee of the Faculty of Social Welfare and Health Sciences at the University of Haifa (approval #118/16). All the study participants gave their written consent to participate in the research and publish its results.

## Results

The factors influencing vaccination uptake were investigated using multiple logistic regression that included the ethnicity factor (Jewish/Arab) along with other factors expected to influence vaccination compliance: perceived risk of influenza, perceived risk of the influenza vaccine, school-located vaccination program, education, and literacy difficulties. Ethnicity was found to be a significant factor (OR = 3.14, 95% CI = [1.90,5.19]), implying that the odds for vaccination uptake in the Arab population are more than three times greater than in the Jewish population. Perceived risk of influenza was found to be significant (OR = 1.75, 95% CI = [1.37,2.24]), implying that an increase of one unit on the perceived disease risk index increases the odds of vaccination uptake by 75%. Perceived vaccine risk was also found to be significant (OR = 0.19, 95% CI = [0.13,0.28]), implying that an increase of one unit on the perceived vaccine risk index decreases the odds of vaccination uptake by over 80%. School-located vaccination as an indicator of vaccine effectiveness and safety was found to be significant (OR = 1.39, 95% CI = [1.16,1.67]), implying that an increase of one unit on the school-located vaccination index increases the odds of vaccination uptake by almost 40%. Difficulties in literacy were found to be significant (OR = 1.43 95% CI = [1.17,1.75]), implying that an increase of one unit on the index of literacy difficulties increases the odds of vaccination uptake by 43%. The factor of education was not found to be significant (OR = 0.90 95% CI = [0.73,1.11]), such that level of education does not affect the odds of vaccination uptake. This may be related to the variable of literacy and information-seeking difficulties, which was found to be negatively correlated with the education variable, such that the higher one’s educational level, the fewer difficulties one will have with literacy and searching.

### Hypothesis 1: Impact of ethnicity on seasonal influenza vaccination uptake

We used Chi square testing to examine differences in vaccination uptake rates between the two groups. A significant difference emerged between the Jewish and the Arab mothers, such that the Arab mothers reported a vaccination rate of 61.7%, significantly higher than the rate of 33.5% reported by the Jewish mothers (χ^2^(1) = 39.15, P<0.0001). Furthermore, we used independent sample t-testing to examine differences in literacy. The findings point to significant differences between the Arab and the Jewish populations (t (691) = -5.81, p < 0.0001) in literacy and in the ability to seek information regarding the seasonal influenza vaccination. In general, mothers in the Arab population have more literacy difficulties and are less able to seek information about the influenza vaccine (M = 3.1545, SD = 1.1478) than mothers in the Jewish population (M = 2.5654, SD = 1.0149).

### Hypothesis 2: Risk perceptions regarding seasonal influenza vaccination

To test this hypothesis, we first calculated Pearson correlation coefficients to examine the correlation between general attitudes toward vaccinations and perceived risk of the influenza vaccination in each of the two groups. After that, we conducted a Fisher correlation comparison test to compare the correlations between the Arab population and the Jewish population with respect to the relationship between attitudes and risk perceptions. The research findings show that in both population groups a significant negative correlation emerged between mothers’ attitudes and their risk perceptions regarding the seasonal influenza vaccination (Jewish group: Pearson correlation coefficient = -0.4329, P < .0001; Arab group: Pearson correlation coefficient = -0.2475, P < .0001). That is, the less positive the mothers’ attitudes toward vaccination in general, the riskier they perceived the influenza vaccination. Moreover, the findings reveal a significant difference (Z = -2.31, P = 0.02) between the Arab and Jewish populations with respect to the correlation between attitudes and vaccination risk perceptions, with a significantly stronger correlation in the Jewish population than in the Arab population.

We used t-testing for independent samples to test the difference in perceived vaccination risk in the two population groups. The results show a significant difference (t (691) = 2.74, p = 0.0063) between the Arab population and the Jewish population in their perceptions of the risks involved in the seasonal influenza vaccination. In general, the Jewish population perceives the vaccination as riskier (M = 3.0696, SD = .8371) than does the Arab population (M = 2.8437, SD = 0.9314).

### Hypothesis 3: Risk perceptions regarding seasonal influenza as a disease

We used t-testing for independent samples to examine disease risk perceptions in the two populations. The results show that the Arab population and the Jewish population do not differ significantly (t (691) = 1.20, df = 691, p = 0.2318) in their perceptions of the risks of seasonal influenza. In other words, perceptions of the severity of the disease among the Arab population (M = 2.7598, SD = 1.0152) are relatively similar to those among the Jewish population (M = 2.8671, SD = 0.8984).

### Hypothesis 4: Inclusion of seasonal flu vaccinations in the school-located vaccination program

To examine the impact of SLIV, we conducted a Wilcoxon test for independent samples. The research findings revealed a significant difference (Z = 6.6719- P < .0001) between the Arab and Jewish populations in how they perceive the impact of SLIV. For the Arab mothers, inclusion of the influenza vaccine in the school-located program provided more legitimization to their decision to vaccinate their children (Wilcoxon mean rank = 372.99) than it did for Jewish mothers (Wilcoxon mean rank = 254.94). This may explain the higher influenza vaccination uptake in the Arab population than in the Jewish population.

## Discussion

To the best of our knowledge, the current study is the first of its kind to examine how inclusion of the seasonal influenza vaccination in the school-located vaccination program influences decisions made by Arab and Jewish mothers regarding influenza vaccination uptake. Moreover, this is the first study to describe maternal decision-making about seasonal influenza vaccination in the 7–9 years age group. The most recent statistics in Israel show that the rate of seasonal influenza vaccination among Arab children aged 7–9 years is significantly higher than among the same age group of Jewish children. Indeed, the vaccination uptake rate among Arab mothers for seasonal influenza vaccination as well as for all other vaccinations is almost twice that of Jewish mothers. Prior research has attempted to explain this finding. One major factors found to influence this high compliance rate is the trust that Arab mothers place in the recommendations of the Tipat Halav (Family Health Center) nurses. Other factors contributing to this high uptake rate include prevalent social norms regarding vaccination in Arab society, the effective influence of Ministry of Health campaigns and compliance with Ministry of Health directives [39, 42, 43]. As in other studies, the findings of this study show that various factors can significantly influence maternal compliance with seasonal influenza vaccination, among them influenza risk perceptions [44], perceived safety and efficacy of the influenza vaccine (fears of side effects) [25, 35], and health literacy and information-searching abilities [45–47].

The current study examined the correlation between attitudes toward vaccination and risk perceptions regarding the seasonal influenza vaccine as well as the impact of this correlation on seasonal influenza vaccination uptake among mothers from different population groups. The findings show that attitudes exert a greater influence on Arab mothers’ vaccine risk perceptions than on those of Jewish mothers, thus encouraging greater vaccination compliance among Arab mothers [39].

Our examination of the differences between the two groups with respect to perceived vaccination risk and perceived illness risk shows that Arab mothers perceive the seasonal influenza vaccination as safer than do Jewish mothers. These findings resemble those of other studies conducted among the Arab population in Israel showing that Arab Muslim parents believe that a vaccine’s effectiveness is greater than its risk, thus apparently encouraging Arab mothers to be more compliant in vaccinating their children [25, 43]. Furthermore, the findings of the current study regarding the perceived risks of influenza indicate that the two groups do not differ in their risk perceptions. These findings differ from those of a previous study (2016) conducted among different populations in Israel showing that Muslim mothers consider seasonal influenza to be a more dangerous illness than do Jewish mothers [25]. In contrast, a 2019 study showed that influenza risk perception has a greater influence on vaccination decision-making in the Jewish population than in the Arab population [45].

Examination of the SLIV variable indicates that the legitimation provided by this inclusion finds expression in higher uptake among Arab mothers than among Jewish mothers [43]. These results can be explained by the cognitive dissonance theory, according to which people aspire to achieve a conscious balance between their opinions and their actions. Hence, they disregard data that are not in line with their views. With respect to the current study, Arab mothers hold positive attitudes towards vaccines in general and therefore see the vaccination as safer and do not consider its potential risks, thus increasing their compliance. In contrast, Jewish mothers have fewer positive attitudes towards vaccines in general and at the same time see the influenza vaccine as less effective, resulting in lower vaccination compliance [48].

The research findings also point to another component that further reinforces the cognitive dissonance theory. According to Heider’s (1946) balance theory, individuals strive to maintain cognitive and emotional balance between their opinions and those of others. In a state of balance, the relations among the three components (individual, other and topic) are positive. A lack of balance leads to discomfort, such that the individual will act to achieve a state of comfort. The three factors in the balance triangle of the current study are: attitudes regarding vaccines in general; perceived risks and safety of the influenza vaccination; and legitimization provided by SLIV. Among Arab mothers, these factors demonstrate a positive balance (positive vaccination attitudes in general (+), low perceived risk of the influenza vaccine (+) and high legitimacy provided by SLIV (+)). In contrast, among Jewish mothers these three factors exhibit a negative balance (negative vaccination attitudes in general (-), high perceived risk of influenza vaccine (-) and only partial legitimacy provided by SLIV (-)). In effect, the situation among Arab mothers is the mirror image of the situation among Jewish mothers.

The differences that emerged between Jewish and Arab mothers in the current study have several possible explanations. First, research indicates that Jewish mothers seek information more actively than do Arab mothers, pointing to a higher level of health literacy among Jewish mothers than among Arab mothers. Hence, Jewish mothers are more capable of looking for and finding information about the influenza vaccination that is not mediated by the health institution, thus exposing them to controversies regarding the seasonal flu vaccine [46, 49].

Second, although education did not significantly affect compliance, this factor was positively correlated with literacy difficulties. Other studies investigating the effect of education on Arab mothers’ uptake of the influenza vaccine found the education factor to exert a significant influence. One study conducted in Israel among Arab mothers showed that education is clearly associated with vaccine uptake, such that the lower the mother’s education, the higher her vaccination compliance [45]. Third, in a previous study we found that explanatory materials targeting the Arab population are not sufficiently culturally appropriate to the level of language and literacy in that society. Therefore, mothers tend to trust the schools more and reason that if the school includes the vaccination in the school-located program, it must be safe, so they tend not to seek information on their own [39, 50]. More specifically, because Arab mothers for the most part do not read the materials distributed by the health authorities as well as due to their objective difficulties in accessing other scientific materials, they may tend to accept the recommendations of the school that legitimize the effectiveness and safety of the flu vaccine.

## Conclusions

This research reiterates the influence of SLIV, particularly among those who already have positive attitudes toward vaccinations. Moreover, low risk perceptions regarding the influenza vaccine and low levels of literacy tend to prevent people from seeking information. Future research should examine the influence of social discourse and information-seeking on parents’ opinions and perceptions and on their compliance with school-located vaccinations. The research also points to the need for education and tools to boost health literacy among minority populations so that mothers can make independent and informed decisions, based on currently available evidence. Moreover, mothers should be provided with up-to-date information that is readily available in the professional literature.

### Limitations

One limitation of this study is that it is based on mothers of children aged 7–9 years who agreed to participate in this research. This may have led to a self-selection bias regarding mothers who volunteered to participate in the study. This type of bias is present in any research based upon voluntary participation. In addition, the choice of mothers and not fathers stemmed from findings that mothers are usually the ones to decide whether their children will be vaccinated, especially in the Arab population [37]. However, the inclusion of both parents (fathers and mothers) might change the presented data or affect the significance of the factors tested if the combined data was analyzed for both parents or the fathers as a distinctive group.

## Supporting information

S1 Appendix Research questionnaire. (PDF)
