# Supplementary material for: Risk perceptions regarding inclusion of seasonal influenza vaccinations in the school immunization program in Israel: Arab vs. Jewish mothers
Source: PLoS One. 2022 Apr 18;17(4):e0267279. doi: 10.1371/journal.pone.0267279 (PMC9015128; doi:10.1371/journal.pone.0267279)
Supplement: S1 Appendix — (PDF) [file pone.0267279.s001.pdf]

# Appendix 1: Research questionnaire

Dear Participant,

This questionnaire is part of a doctoral research project at the School of Public Health at the University of Haifa.

The research examines **mothers' decision-making processes regarding seasonal influenza vaccination uptake**

The required research population includes mothers who have young children eligible to receive the seasonal influenza vaccination at school.

The questionnaire is user-friendly and research participation is anonymous, without any identifying details.

The information provided will be used for research purposes only and under no circumstances will be given to any other person or organization.

Please note that there are no right or wrong answers. The correct answer is the one that reflects your personal opinion.

I will be very grateful if you can help by answering the following questions. Your responses will make a major contribution to the research.

Thank you in advance for your cooperation and willingness to participate in the research.

The Research Team:

Nour Abed Elhadi Shahbari, Prof. Anat Gesser-Edelsburg, Prof. Gustavo Mesch

**Please answer the following questions:**

- Age ________
- Number of children ______
- Mother's education:

  1. Elementary 2. Secondary 3. Post-secondary 4. Bachelor's degree

  5. Master's degree and higher
- Family income:

  1. 2500-4000 2. 4000-6500 3. 6500-8000 4. 8000-12000 5. 12,000 and above
- Residential area:

  1. City 2. Village 3. Moshav 4. Kibbutz
- Degree of religious observance:

  1. Secular 2. Traditional 3. Religious
- Ethnicity

  1. Jewish 2. Muslim 3. Christian 4. Druse 4. Bedouin

**Please answer the following questions:**

- Do you vaccinate your children according to the schedule dictated by Tipat Halav and the Ministry of Health?

  1. Yes 2. Partially 3. Do not vaccinate

**Rate the extent of your agreement with the following statements:**

| | Do not agree at all | Agree to a small extent | Agree to a moderate extent | Agree to a high extent | Agree to a very high extent |
|---|---|---|---|---|---|
| All vaccinations recommended by the Ministry of Health are safe | 1 | 2 | 3 | 4 | 5 |
| All vaccinations recommended by the Ministry of Health are effective | 1 | 2 | 3 | 4 | 5 |
| When I think about vaccinations I feel safe | 1 | 2 | 3 | 4 | 5 |
| When I think about vaccinations I am afraid of side effects | 1 | 2 | 3 | 4 | 5 |
| When I vaccinate my children I feel calm | 1 | 2 | 3 | 4 | 5 |
| When I don’t vaccinate my children I have feelings of guilt | 1 | 2 | 3 | 4 | 5 |
| I trust the health system in Israel because of the quality of care and the service | 1 | 2 | 3 | 4 | 5 |
| I do not trust the health system in Israel but I have no choice but to depend on it | 1 | 2 | 3 | 4 | 5 |

| | | | | | |
|---|---|---|---|---|---|
| I trust my family doctor when it comes to vaccinations because he is an expert and knows more than I do | 1 | 2 | 3 | 4 | 5 |
| I do not always believe my family doctor and I check the medical information he gives me about vaccinations | 1 | 2 | 3 | 4 | 5 |
| I don’t have time to look for information about vaccinations so I make do with the information I receive from the medical team (nurse and doctor) | 1 | 2 | 3 | 4 | 5 |
| I have difficulty understanding written materials in English that provide information about vaccinations | 1 | 2 | 3 | 4 | 5 |
| I feel I lack the skills and abilities to search the internet and to be able to determine what is reliable information about vaccinations | 1 | 2 | 3 | 4 | 5 |

**Vaccination against seasonal influenza:**

- Did you vaccinate your child against seasonal influenza this year?

  1. Yes 2. No

If no, why not? ________________________________________

If yes, why? ___________________________________________

**<u>Rate the extent of your agreement with the following statements</u>:**

| | Do not agree at all | Agree to a small extent | Agree to a moderate extent | Agree to a high extent | Agree to a very high extent |
|---|---|---|---|---|---|
| Seasonal influenza is a very serious illness | 1 | 2 | 3 | 4 | 5 |
| Seasonal influenza is an illness that the body can cope with and does not require a vaccination | 1 | 2 | 3 | 4 | 5 |
| The seasonal influenza vaccination is safe | 1 | 2 | 3 | 4 | 5 |
| The seasonal influenza vaccination is effective | 1 | 2 | 3 | 4 | 5 |
| I fear the side effects of the seasonal influenza vaccination | 1 | 2 | 3 | 4 | 5 |
| I vaccinate my son/daughter based on the recommendation of family/friends | 1 | 2 | 3 | 4 | 5 |

| | | | | | |
|---|---|---|---|---|---|
| I do not have enough information about the seasonal influenza vaccination to make a decision | 1 | 2 | 3 | 4 | 5 |
| The moment the seasonal influenza vaccination was introduced to the school-located program, my acceptance of the vaccination became higher | 1 | 2 | 3 | 4 | 5 |
